# Reproductive Status Alters Transcriptomic Response to Infection in Female *Drosophila melanogaster*

**DOI:** 10.1534/g3.112.005306

**Published:** 2013-05-01

**Authors:** Sarah M. Short, Brian P. Lazzaro

**Affiliations:** Field of Genetics and Development, Cornell University, Ithaca, New York 14853

**Keywords:** reproduction, immunity, microarray, gene expression, *Drosophila melanogaster*

## Abstract

Mating and consequent reproduction significantly reduce the ability of female *Drosophila melanogaster* to defend against systemic bacterial infection. The goal of the present study was to identify genes likely to inform the mechanism of this post-mating immunosuppression. We used microarrays to contrast genome-wide transcript levels in virgin *vs.* mated females before and after infection. Because the immunosuppressive effect of mating is contingent on the presence of a germline in females, we repeated the entire experiment by using female mutants that do not form a germline. We found that multiple genes involved in egg production show reduced expression in response to infection, and that this reduction is stronger in virgins than it is in mated females. In germline-less females, expression of egg-production genes was predictably low and not differentially affected by infection. We also identified several immune responsive genes that are differentially induced after infection in virgins *vs.* mated females. Immune genes affected by mating status and egg production genes altered by infection are candidates to inform the mechanism of the trade-off between mating and immune defense.

Although our knowledge of the invertebrate immune system is extensive and continues to expand (reviewed in Wang and Ligoxygakis 2006; Lemaitre and Hoffmann 2007), our understanding of overall immune defense remains incomplete. Overall immune defense is defined as the combined abilities to immunologically eliminate pathogens and to tolerate the damage associated with an infection (Råberg *et al.* 2009). Part of our lack of understanding of immune defense stems from the fact that defense is not determined only by immune system activity but is also influenced by aspects of host physiology outside the canonical immune system. These nonimmunological processes are often responsive to environmental factors such as temperature, nutritional availability, or interactions with other organisms. The interconnection of defense with other diverse aspects of host physiology can set the stage for trade-offs between immunity and other costly life-history traits (Lazzaro and Little 2009; Parker *et al.* 2011). Trade-offs between life-history traits and immunity have the potential to limit the evolution of immune efficacy, and their study forms the basis of the emerging field of ecological immunology (Sheldon and Verhulst 1996; Siva-Jothy *et al.* 2005; Schulenburg *et al.* 2009).

Studies in ecological immunology have typically focused on identifying trade-offs between immune defense and life history traits, yielding remarkable progress in our understanding of immune defense in ecological and evolutionary contexts. Less emphasis has been placed on determining the mechanistic nature of these trade-offs, and our lack of mechanistic understanding represents a significant gap in our understanding of the function of immune defense (Schmid-Hempel 2003). We and others have demonstrated that mated females suffer reduced ability to eliminate and survive pathogenic infection relative to virgin females (Fedorka *et al.* 2007; Short and Lazzaro 2010; Short *et al.* 2012). We have also shown that the effect of mating on immune defense is contingent on the proper formation of the female germline (Short *et al.* 2012), suggesting that post-mating immunosuppression is dependent on an as-yet unknown aspect of reproduction. The objective of the present study was to use transcriptional profiling to begin to identify why mated females demonstrate reduced immune defense. To address this question, we used whole-genome microarrays to test for differences in the transcriptional response of virgin females to systemic bacterial infection as compared to the response of mated females. We also sought to determine how infection status alters transcript levels of mating-responsive genes. We then repeated this experiment in females who failed to form a germline to determine which changes in gene expression were likely to be genetically or physiologically related to egg production. Our goal was to identify transcriptional processes that are most likely to be involved in shared signaling between immunity and reproduction and thus most likely to underlie the observed trade-off.

## Materials and Methods

### Fly stocks and maintenance

Female flies used in this experiment were derived from two crosses: egg-producing females were *tud^1^ bw sp/*CS and were the daughters of a cross between *tud^1^ bw sp/*CyO mothers and Canton-S fathers. Eggless females were also *tud^1^ bw sp/*CS but were the daughters of *tud^1^ bw sp* mothers and Canton-S fathers. The mothers of the eggless females were homozygous for *tudor^1^*, a recessive maternal effect mutation that causes offspring to lack pole cells and therefore fail to form a germline. Egg-producing females had a genotype identical to eggless females but because their mothers were heterozygous for *tudor^1^*, they produced normal numbers of eggs. Males used in mating experiments were from the standard laboratory strain Canton-S.

### Mating procedure

Eggless and egg-producing females were collected as virgins and aged for 3 d post-eclosion. The day before matings were to be set up, eggless and egg-producing females were lightly anesthetized with CO_2_ and put into individual vials with *ad libitum* access to food (8.3% glucose, 8.3% brewer’s yeast, and 1% agar, plus 0.04% phosphoric acid and 0.4% propionic acid added to inhibit microbial growth in the food). Females were randomly allocated to “virgin” or “mated” treatment groups and allowed to recover overnight. The following morning, within 2 hr of incubator “dawn,” a single virgin male was aspirated into each vial containing a female assigned to the “mated” treatment and individual copulations were observed. Males were removed from the presence of females shortly after copulation cessation to prevent additional courting or copulation attempts. Egg-producing females mated for an average of 23.8 min and eggless females mated for an average of 22.6 min. Females from copulations lasting fewer than 15 min were discarded and not used for infections to maximize the likelihood that all females used in the experiment received a full complement of sperm and seminal fluid from their mates.

### Infection procedure and sample preparation

At 2.5 hr (±15 min) after mating, mated eggless and egg-producing females were lightly anesthetized with CO_2_ and infected; age- and rearing-matched virgin controls were infected in parallel. We have previously shown that females are already immunocompromised by 2.5 hr after mating (Short and Lazzaro 2010; Short *et al.* 2012), but this is too soon for many other direct consequences of egg production to manifest. It therefore is an appropriate time at which to measure rapid changes in female condition. Infections were performed by dipping a 0.15-mm anodized steel needle (Fine Science Tools, Inc.) into a dilute bacterial culture of the Gram-negative bacterial pathogen *Providencia rettgeri*, then piercing the thorax of the female fly. *P. rettgeri* was grown with shaking overnight in liquid Luria-Bertani (LB) broth at 37°, then diluted in sterile LB to an optical density of A_600_ = 1.0 ± 0.05. In parallel, females to remain as uninjured controls were anesthetized on CO_2_ to control for effects of anesthesia. Infected mated and virgin females as well as uninjured virgin and mated controls were then put on fresh media in groups of approximately 10. We used uninjured controls in our experiment to detect the combined effects of both septic wounding and the presence of bacteria. A small number of flies were individually homogenized immediately after each round of infection, and an aliquot of undiluted homogenate was quantitatively plated on LB agar using a spiral plater (Microbiology International). We found that our infection technique delivered an average dose of 1.4 × 10^3^ (standard error = 4.7 × 10^2^) bacteria to each female. We have found that the bacterial load of mated females begins to deviate from that of virgins at approximately 12 hr after infection (Supporting Information, Figure S1) (Short *et al.* 2012). We assayed for transcriptomic differences shortly before this point to detect genes potentially responsible for the divergence. Ten hours (±15 min) after infection (approximately 12.5 hr after mating), 25 whole female flies from each treatment were collected on CO_2_, snap frozen in TRIZOL reagent (Ambion), and placed at -80°. The entire experimental set up was replicated on three independent days, resulting in three biological replicates for each of the eight experimental groups.

### RNA extraction and microarray preparation

We extracted RNA from our samples using TRIZOL reagent according to the manufacturer’s protocol. Residual genomic DNA contamination was removed using TURBO DNA-*free* (Ambion), and the quality of the RNA from each sample was assessed using a BioAnalyzer 2100 (Agilent). The BioAnalyzer outputs for our samples showed strong, distinct peaks corresponding to 18S and 28S rRNA with little to no baseline signal between these peaks. This indicated that our samples were high quality with little degradation. Samples were labeled using Agilent’s Low Input Quick Amp Labeling kit and were hybridized to 4x44K *Drosophila* (V2) Gene Expression Microarrays (Agilent) according to the manufacturer’s instructions. RNA labeling, microarray hybridizations and feature extraction were performed by the Cornell University Life Sciences Core Laboratory Center.

### Microarray data analysis

**Figure 1:**
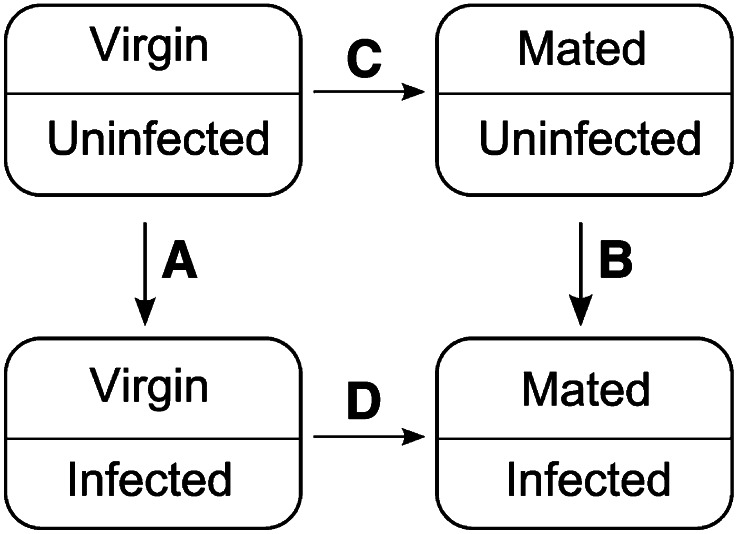
Experimental design. To determine ways in which immune defense and reproduction may interact to cause post-mating immunosuppression, we compared genome-wide transcript abundance between virgin and mated, infected and uninfected females. In each contrast, the arrow conveys the treatment−control relationship, with the arrow emanating from the “control” condition and pointing toward the “treatment” condition in each analysis. We assayed for differential transcript abundance between virgin uninfected females and virgin infected females to identify infection-responsive genes in virgins (A) or mated females (B). By qualitatively comparing (A) with (B), we were able to establish differences in infection response that were dependent on mating status. By subtracting (A) from (B), we were able to ascertain which genes showed the largest quantitative differences in infection response between virgin and mated females. We also assayed for differential transcript abundance between virgin *vs.* mated females when infected (C) or uninfected (D) to determine which genes respond to mating and which differences depend on infection status. We independently performed this entire experimental design in triplicate for both egg-producing females and eggless females.

The microarray data were analyzed using the Bioconductor package limma (Smyth 2005). Data were background corrected by using backgroundCorrect() and the “normexp” method recommended by Ritchie *et al.* (2007). We then normalized between all egg-producing arrays and between all eggless arrays by using quantile normalization as recommended by Agilent, averaging signals between replicate probes. We generated lists of differentially expressed probesets using the method for factorial designs outlined by Smyth (2005). We assayed for gene expression differences due to infection in both virgin and mated females as well as differences due to mating in both uninfected and infected females (Figure 1; Table S1 and Table S2). We also assayed for genes that showed a significant interaction between mating status and infection status (Table S3 and Table S4). These contrasts were initially performed within treatments of egg-producing females and then were separately repeated for arrays from eggless females. Many genes on the 4x44K *Drosophila* (V2) Gene Expression Microarrays (Agilent) were represented by multiple probesets with distinct probe sequences. We performed our analyses at the probeset level and report all difference values for all probesets in Table S1 and Table S2. For simplicity, we present results at the level of gene rather than probeset in the text. In figures, when more than one probeset showed significantly altered expression for a particular gene, we report the probeset with the largest fold change.

We corrected for multiple tests using the Benjamini-Hochberg method (Benjamini and Hochberg 1995) with a false discovery rate of 5.0%. Finally, we validated the gene identities in our lists of differentially expressed probes and eliminated those that did not have an identifiable gene name or gene symbol on Flybase (www.flybase.org). Hypergeometric tests for enrichment of genes with shared Biological Process Gene Ontology (GO) terms were performed using the online tool GOrilla (Eden *et al.* 2009). REVIGO (Supek *et al.* 2011) was used to eliminate redundant GO terms, and multiple-test correction for significant GO terms was performed using the Benjamini-Hochberg method (Benjamini and Hochberg 1995) with a false discovery rate of 5.0%. A subset of results from the microarray experiments were validated using quantitative real-time polymerase chain reaction as described in File S1, Figure S2, Figure S3, Table S5, Table S6, and Table S7.

## Results and Discussion

We sought to identify transcriptional processes that may illuminate the nature of the reduction in immune defense suffered by *D. melanogaster* females after mating. We infected mated, egg-producing females at 2.5 hr after the cessation of copulation alongside virgin, egg-producing controls with the Gram-negative bacterial pathogen *Providencia rettergi*. Ten hours after infection, we assayed genome-wide transcript abundance in infected virgin and mated females as well as in uninfected, age-matched virgin and mated females (Figure 1). We then assayed for genes that showed infection-induced changes in transcript abundance in virgin and/or mated females (comparisons A and B in Figure 1). We also assayed for genes that showed mating-induced changes in transcript abundance in uninfected and/or infected females (comparisons C and D in Figure 1). Results of all treatment comparisons for all probesets for egg-producing females can be found in Table S1. We replicated the entire experiment using females that genetically fail to form a germline to identify transcriptional differences that depend on the presence of a germline. Results of all treatment comparisons for all probesets for eggless females can be found in Table S2. We chose to assay transcript levels at 10 hr after infection because mated females begin to demonstrate higher bacterial loads than virgins at approximately 12 hr after infection (Figure S1) (Short *et al.* 2012) and we were interested in identifying differences in transcript abundance that have the potential to indicate mechanisms for this initial post-mating divergence in immune defense.

### General expression response of females after bacterial infection

#### In egg-producing females:

**Figure 2:**
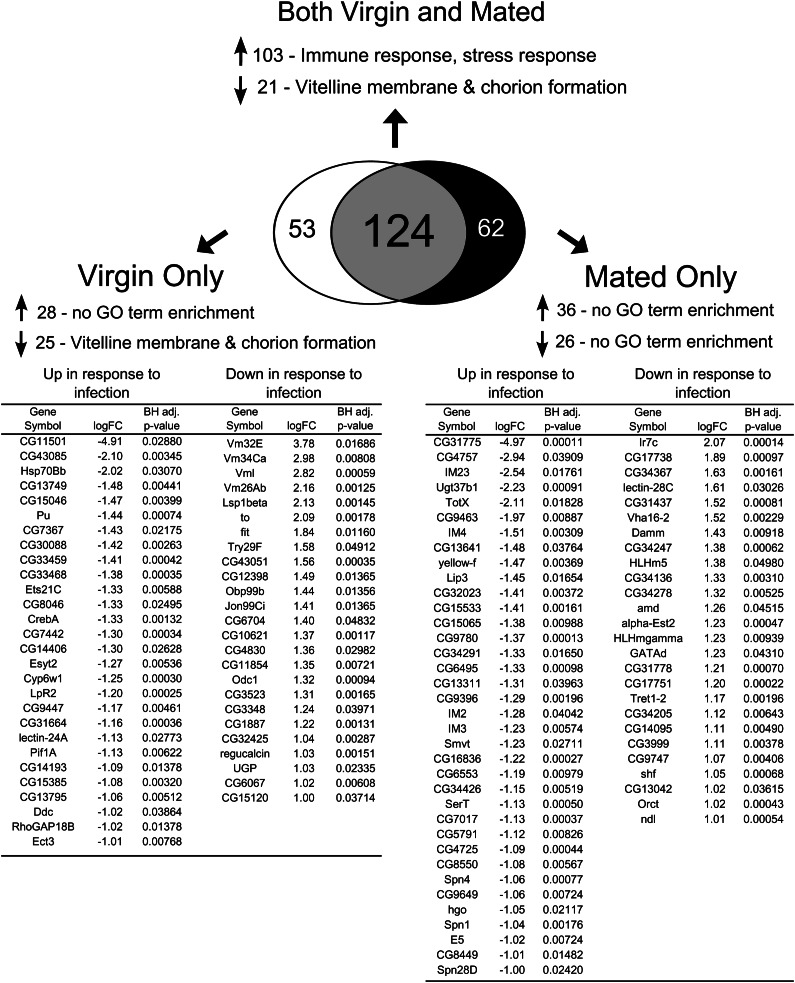
The effect of infection on transcript abundance in virgin and mated females. We assayed for genes that exhibited statistically significant 2-fold or greater differences in transcript abundance in virgin uninfected *vs.* virgin infected treatments and in mated uninfected *vs.* mated infected treatments. We then determined which genes change significantly in transcript abundance due to infection in both virgin and mated females, only in virgins, or only in mated females. Fold change values are in log_2_ units and are expressed as uninfected minus infected signal; therefore, a negative logFC represents increased expression in response to infection whereas a positive logFC represents decreased expression in response to infection. In instances in which more than one probe showed significantly altered expression for a particular gene, only the probeset with the largest fold change is listed. GO term enrichment was determined using GOrilla and REVIGO was used to reduce lists of GO terms to those least redundant. Upward-pointing arrows indicate genes with increased expression and downward-pointing arrows indicate genes with depressed expression. A Benjamini-Hochberg correction (Benjamini and Hochberg 1995) was performed to correct for multiple tests, and only GO terms that were significant after controlling for a false-discovery rate of 5% were retained.

By examining gene expression changes that occur in response to infection in both virgin (comparison A, Figure 1) and mated females (comparison B, Figure 1), we could determine a general infection response profile of female *Drosophila melanogaster* that was consistent across different reproductive states. We detected significant expression changes as a result of bacterial infection in both virgin and mated females in 124 genes (Figure 2, Table S1). Of these 124 genes whose expression changed in response to infection, 103 were up-regulated. Most of these genes are known immunity genes (Figure 2, Table S1).

When we assigned GO terms to the genes up-regulated after infection in both virgin and mated females, we found enrichment of multiple GO terms relating to immune response and stress response (Table 1). As expected, transcript abundance of antimicrobial peptide genes was dramatically increased due to infection (*CecA1*, *CecA2*, *CecB*, *AttA*, *AttB*, *AttC*, *AttD*, *Dpt*, *DptB*, *Mtk*, *Def*, *Dro*, *Drs*, *Drs-l*; Table S1), as was that of many peptidoglycan recognition proteins (*PGRP-SA*, *PGRP-SB1*, *PGRP-SB2*, *PGRP-SC2*, *PGRP-SD*, *PGRP-LB*, *PGRP-LC*, *PGRP-LF*; Table S1). We also found infection-induced increases in transcript abundance in multiple genes in the *Turandot* gene family (*TotA*, *TotB*, *TotC*, and *TotM*; Table S1). At least one gene in the *Tot* family (*TotA*) is regulated by the JAK/STAT signaling pathway (Agaisse *et al.* 2003; Agaisse and Perrimon 2004). Notably, *Tot* genes also respond to general stress conditions (Ekengren and Hultmark 2001), and they may alter immune defense through stress-response mechanisms such as tissue repair. Considering that our infection procedure involved wounding the fly, it is possible that expression changes in these genes reflect a response to injury rather than infection. Other up-regulated genes that are known to respond to infection included *TepII*, *IM3*, *IM1*, *IM10*, *Rel*, *pirk*, *spirit*, *edin*, *TsfI*, and *nimB1* (Table S1). We note that some of the genes we detected as being up-regulated have negative regulatory roles in the humoral immune response (*PGRP-LB*, *PGRP-SC2*, *pirk*), illustrating mechanisms by which the host modulates immune system activity (Paredes *et al.* 2011).

**Table 1:** Biological process information for genes significantly altered by infection in virgin and/or mated egg-producing females

Gene List	GO Term	GO Term Description	Corrected *P* Value	No. Genes in GO Category
Up significantly after infection in both virgin and mated females	GO:0009617	Response to bacterium	2.01E-38	31
	GO:0006952	Defense response	2.65E-37	35
	GO:0009607	Response to biotic stimulus	5.01E-34	32
	GO:0051704	Multiorganism process	7.76E-30	33
	GO:0006955	Immune response	5.07E-29	28
	GO:0002376	Immune system process	4.03E-27	28
	GO:0006950	Response to stress	4.96E-26	41
	GO:0009253	Peptidoglycan catabolic process	8.26E-12	8
	GO:0050896	Response to stimulus	2.91E-11	45
	GO:0030203	Glycosaminoglycan metabolic process	3.23E-09	8
	GO:0016052	Carbohydrate catabolic process	5.56E-07	9
	GO:0005976	Polysaccharide metabolic process	1.71E-05	10
	GO:0031347	Regulation of defense response	8.52E-05	6
	GO:0034605	Cellular response to heat	9.98E-05	5
	GO:0009308	Amine metabolic process	5.53E-04	12
	GO:0005975	Carbohydrate metabolic process	6.19E-04	13
	GO:0043900	Regulation of multiorganism process	6.32E-04	6
	GO:0009595	Detection of biotic stimulus	1.22E-03	3
	GO:0009057	Macromolecule catabolic process	1.91E-03	9
	GO:0080134	Regulation of response to stress	3.45E-03	6
	GO:0034644	Cellular response to UV	8.01E-03	3
	GO:0008063	Toll signaling pathway	1.29E-02	4
	GO:0009266	Response to temperature stimulus	1.39E-02	6
	GO:0061060	Negative regulation of peptidoglycan recognition protein signaling pathway	1.81E-02	2
	GO:0071214	Cellular response to abiotic stimulus	3.19E-02	3
	GO:0009411	Response to UV	3.67E-02	3
Down significantly after infection in both virgin and mated females	GO:0007305	Vitelline membrane formation involved in chorion-containing eggshell formation	1.04E-03	3
	GO:0022412	Cellular process involved in reproduction in multicellular organism	1.63E-03	4
	GO:0010927	Cellular component assembly involved in morphogenesis	3.51E-03	4
	GO:0043062	Extracellular structure organization	1.75E-02	3
Up significantly after infection in only virgin females	No enrichment			
Down significantly after infection in only virgin females	GO:0007305	Vitelline membrane formation involved in chorion-containing eggshell formation	1.75E-05	4
	GO:0043062	Extracellular structure organization	9.23E-04	4
	GO:0022412	Cellular process involved in reproduction in multicellular organism	1.08E-03	4
	GO:0010927	Cellular component assembly involved in morphogenesis	1.02E-02	4
Up significantly after infection in only mated females	No enrichment			
Down significantly after infection in only mated females	No enrichment			

GO, Gene Ontology; UV, ultraviolet.

Twenty-one genes showed reduced transcript abundance after infection in both virgins and mated females (Figure 2, Table S1). Notably, this set was enriched for genes involved in egg formation, specifically vitelline membrane and chorion formation (*Vm26Ac*, *Vml*, *psd*, and *dec-1*, Table 1, Table S1). Given that female *D. melanogaster* suffer a germline-dependent reduction in immune defense after mating (Short *et al.* 2012), a generalized decrease in transcription of genes crucial for oogenesis is consistent with a scenario in which reproduction and immune defense are physiologically at odds.

#### In germline-less females:

**Figure 3:**
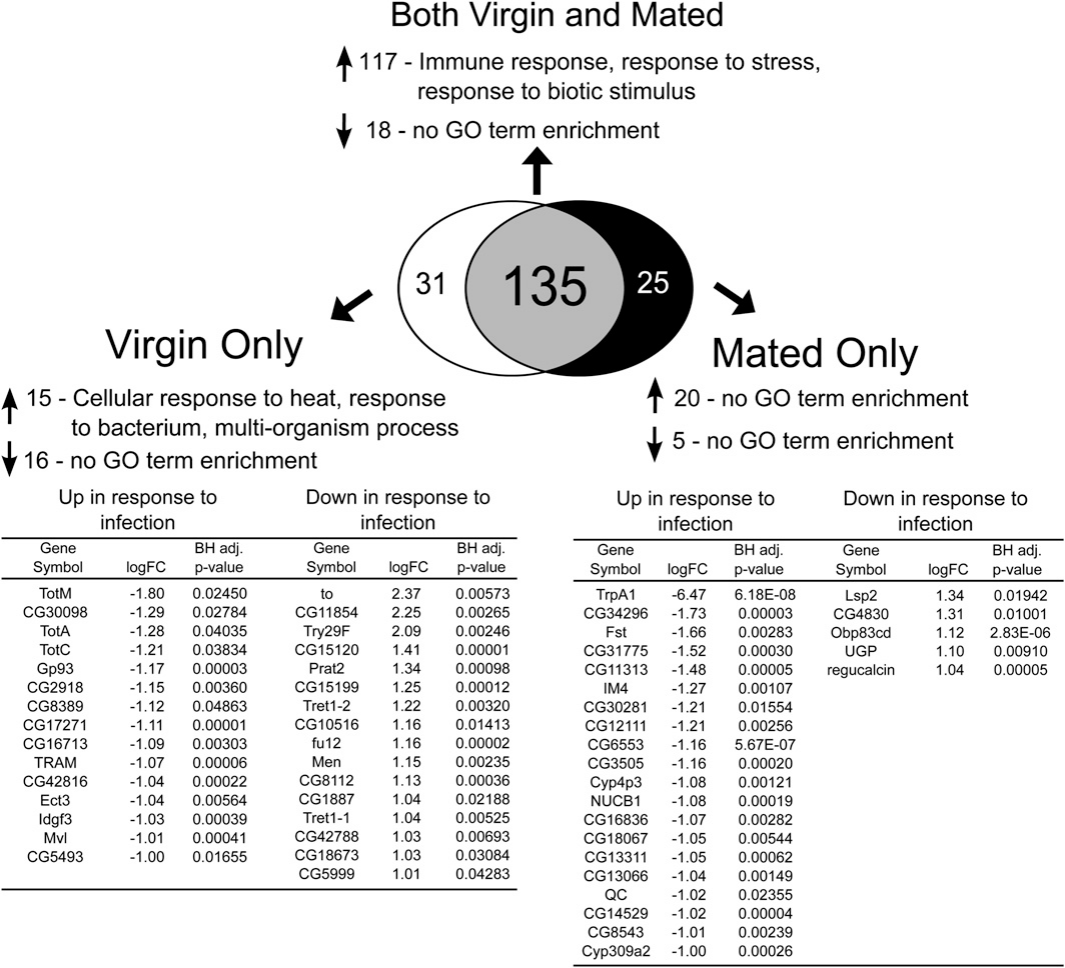
The effect of infection on transcript abundance in virgin and mated eggless females. We assayed for genes that exhibited significant 2-fold or greater differences in transcript abundance in virgin uninfected *vs.* virgin infected treatments and in mated uninfected *vs.* mated infected treatments. We then determined which genes change significantly in transcript abundance due to infection in both virgin and mated females, only in virgins, or only in mated females. Fold change values are in log_2_ units, and are expressed as uninfected minus infected signal; therefore, a negative logFC represents increased expression in response to infection whereas a positive logFC represents decreased expression in response to infection. In instances in which more than one probe for a particular gene showed significant change in expression, only the probeset with the largest fold change is listed. GO term enrichment was determined using GOrilla and REVIGO was used to reduce lists of GO terms to those least redundant. Upward-pointing arrows indicate genes with increased expression and downward-pointing arrows indicate genes with depressed expression. A Benjamini-Hochberg correction (Benjamini and Hochberg 1995) was performed to correct for multiple tests, and only GO terms that were significant after controlling for a false-discovery rate of 5% were retained.

We found that both virgin and mated eggless females shared increased expression of 117 genes and decreased expression of 18 genes in response to infection (Figure 3, Table S2). As was the case for females with intact germlines, the genes whose expression increased in response to infection included many known immunity genes, such as those encoding antimicrobial peptides (*AttA*, *AttB*, *AttC*, *AttD*, *CecA1*, *CecA2*, *Cec2*, *CecB*, *CecC*, *Def*, *Dpt*, *DptB*, *Dro*, *Drs*, *Drs-l*), peptidoglycan recognition proteins (*PGRP-LB*, *PGRP-LC*, *PGRP-LF*, *PGRP-SA*, *PGRP-SB1*, *PGRP-SB2*, *PGRP-SC2*, *PGRP-SD*), and other known infection responsive genes (*edin*, *IM1*, *IM10*, *IM18*, *IM2*, *IM23*, *IM3*, *IM4*, *spirit*, *nimB1*, *Rel*, *TepII*, *TsfI*, *pirk*; Table 2, Table S2). Thus, the general response to infection is not germline dependent. Notably missing from this list, however, are the *Tot* genes. More detailed inspection revealed that expression of *TotA*, *TotC*, and *TotM* increases significantly after infection in virgin but not mated eggless females (Figure 3). This finding is in contrast to egg-producing females, where both virgin and mated females showed significant increases in *Tot* gene expression after infection. These data suggest that infection-induced changes in the expression of *Turandot* genes may be partly germline dependent and that differences in *Tot* inducibility between virgin and mated females may be mediated by the germline.

**Table 2:** Biological process information for genes significantly altered by infection in virgin and/or mated eggless females

Gene list	GO Term	GO Term Description	Corrected *P* Value	No. Genes in GO Category
Up significantly after infection in both virgin and mated females	GO:0006952	Defense response	3.97E-39	38
	GO:0042742	Defense response to bacterium	3.13E-33	28
	GO:0006955	Immune response	8.09E-30	30
	GO:0009607	Response to biotic stimulus	1.03E-28	30
	GO:0002376	Immune system process	5.84E-28	30
	GO:0006950	Response to stress	2.67E-27	45
	GO:0051704	Multiorganism process	8.50E-25	31
	GO:0050896	Response to stimulus	2.97E-12	51
	GO:0009253	Peptidoglycan catabolic process	2.68E-11	8
	GO:0030203	Glycosaminoglycan metabolic process	1.09E-08	8
	GO:0016052	Carbohydrate catabolic process	2.12E-06	9
	GO:0005976	Polysaccharide metabolic process	6.97E-06	11
	GO:0009308	Amine metabolic process	5.12E-04	13
	GO:0043900	Regulation of multiorganism process	1.54E-03	6
	GO:0008063	Toll signaling pathway	1.59E-03	5
	GO:0035079	Polytene chromosome puffing	1.86E-03	3
	GO:0035080	Heat shock-mediated polytene chromosome puffing	1.91E-03	3
	GO:0009595	Detection of biotic stimulus	1.97E-03	3
	GO:0005975	Carbohydrate metabolic process	2.85E-03	13
	GO:0009057	Macromolecule catabolic process	5.61E-03	9
	GO:0080134	Regulation of response to stress	7.47E-03	6
	GO:0061060	Negative regulation of peptidoglycan recognition protein signaling pathway	2.45E-02	2
	GO:0009056	Catabolic process	4.90E-02	12
Down significantly after infection in both virgin and mated females	No enrichment			
			
Up significantly after infection in only virgin females	GO:0034605	Cellular response to heat	1.81E-02	3
	GO:0009617	Response to bacterium	3.28E-02	4
	GO:0051704	Multiorganism process	3.10E-02	5
Down significantly after infection in only virgin females	No enrichment			
Up significantly after infection in only mated females	No enrichment			
Down significantly after infection in only mated females	No enrichment			

GO, Gene Ontology; UV, ultraviolet.

### The effect of mating status on expression of infection-responsive genes

We and others have shown that mated females suffer reduced ability to defend against systemic infection relative to virgin females (Fedorka *et al.* 2007; Short and Lazzaro 2010; Short *et al.* 2012), and this effect is eliminated when the females fail to produce eggs. We hypothesized that virgin females may exhibit gene expression differences after infection that differ from those of mated females, which could inform the nature of the physiological trade-off we have observed between reproduction and immune defense. Changes seen in egg-producing females that are not observed in eggless females may indicate germline-dependent elements of the trade-off.

There were 53 genes whose expression was significantly affected by infection in virgin but not mated females (comparison A but not comparison B in Figure 1; Figure 2). Of these 53 genes, 28 of them were up-regulated by infection, whereas 25 of them were down-regulated. GO analysis on the genes corresponding to up-regulated probesets revealed no enrichment of particular biological processes (Figure 2, Table 1). However, genes involved in vitelline membrane and egg coat formation were enriched within the group of down-regulated genes (Figure 2, Table 1). This enrichment was primarily due to virgin-specific reductions in transcript abundance for the genes *Vm32E* (down 13.74-fold), *Vm34Ca* (down a maximum of 7.89-fold), *Vml* (down 7.06-fold), and *Vm26Ab* (down 4.47-fold) (Figure 2, Table S1). These data suggest that nonreproductive (*i.e.*, virgin) females preferentially suppress expression of genes in egg formation when faced with systemic bacterial infection. These genes are not significantly affected by infection in mated females (with the exception of one probeset for *Vml*; Table S1), likely because mated females continue to produce mature eggs even while combating infection (McKean *et al.* 2008).

We performed a reciprocal analysis to identify changes in gene expression in response to infection that were significant only in mated females but not in virgins (significant in comparison B but not in comparison A in Figure 1). We found 62 genes whose expression was significantly altered by infection in mated females only (Figure 2). Of these 62 genes, 36 were up-regulated by infection whereas 26 were down-regulated (Figure 2). We found no GO categories enriched within either the up-regulated or down-regulated genes, nor in the entire set of 62 genes (Table 1). Nonetheless, we note that expression of multiple genes that have previously been shown to be induced by infection were significantly increased in response to infection in mated females but not in virgins, including *IM2*, *IM3*, *IM4*, and *IM23* (Uttenweiler-Joseph *et al.* 1998), and also *yellow-f* (De Gregorio *et al*, 2001) (Figure 2). This was somewhat surprising given that mated females have lower immune defense than virgin females. At 10 hr after infection, when we assayed gene expression, mated females did not have greater levels of bacteria than virgin controls (Figure S1); therefore, we think it is unlikely that this higher immune gene transcript abundance reflects increased positive stimulation of the immune system through higher pathogen load.

We performed these same analyses in eggless females (Figure 3) and found multiple instances in which gene expression changes differed from those of egg-producing females (Figure 2). We found that eggless females show a virgin-specific increase in genes enriched for “cellular response to heat,” “response to bacterium,” and “multiorganism process” (Figure 3, Table 2). Enrichment of these GO categories can be attributed to virgin-specific changes in *Tot* gene expression, as described previously (Figure 3). In addition, eggless females predictably do not show altered expression of genes encoding vitelline membrane or chorion proteins after infection regardless of mating status (Figure 3, Table S2). This is not unexpected because the germline-less females do not produce eggs, but it does provide a clear example of a germline-dependent difference in the transcriptional response to infection of virgin and mated females. This finding is consistent with our model that post-mating suppression of immune defense is related to energetic expenditure on the production of fertile eggs (Short *et al.* 2012), and a logical extension is that females who produce proportionally more eggs may suffer immunologically to a greater degree.

In addition to querying probesets that were significantly altered by infection in one mating status but not the other, we were also interested in identifying probesets that differed quantitatively in the degree to which expression changed between virgin and mated females. We first assessed this in egg-producing females by identifying genes for which the absolute value of comparison A (Figure 1) minus comparison B (Figure 1) was greater than 1.0, indicating at least a 2-fold difference in response to infection in virgins *vs.* mated females (Table S3). There were 335 genes that met this criterion. We found that for 68 of these genes, the virgin response to infection was significantly different from the mated response to infection at a nominal (uncorrected) p-value of 0.05 (Table S3). GO analysis of the 335 genes showed significant enrichment for four Biological Process terms: “defense response to gram-positive bacterium,” “defense response,” “ATP biosynthetic process,” and “vitelline membrane formation involved in chorion-containing eggshell formation” (Table 3).

**Table 3:** Biological process information for genes showing change in transcript levels due to infection that differ by 2-fold or greater in virgin *vs.* mated egg-producing females

GO Term	GO Term Description	Corrected *P* Value	Genes in GO Category
GO:0007305	Vitelline membrane formation involved in chorion-containing eggshell formation	4.03E-04	Vm26Aa, Vm26Ab, Vml,
			Vm34Ca, Vm32E, closca
GO:0050830	Defense response to Gram-positive bacterium	2.82E-03	sphinx2, AttA, AttB, AttC,
			AttD, PGRP-SD, TotM
			CG30098
GO:0006754	ATP biosynthetic process	2.63E-02	Ca-P60A, CG17300, CG5389,
			ATPsyn-gamma, CG12027, ATP7
GO:0006952	Defense response	4.51E-02	sphinx2, IM4, r2d2, CG30098,
			PGRP-SD, AttA, AttB, AttC,
			AttD, Gr28b, TepII, Eig71Eg,
			TotM, Tsf1

GO, Gene Ontology.

Multiple genes implicated in immune defense were differentially affected by infection in virgins compared with mated females (Table 3). The transcript level of *TepII* is significantly greater after infection in virgins relative to mated females (*P* < 0.05, Table S3). All of the *Attacin* genes and *TotM* are also more strongly induced in virgin females relative to mated females, although not significantly so (Table S3). *PGRP-SD* and *IM4* show significantly greater expression in mated females than in virgins (*P* < 0.05 in both cases, Table S3), whereas *sphinx2*, *r2d2*, and *Gr28b* are increased in response to infection in mated females but decreased in virgins (*r2d2 P* < 0.05, Table S3). These data reveal that virgins respond differently to infection than do mated females, although the differences are complex. The *Attacin* genes and *TepII*, which are induced to a greater degree in virgins, are directly involved in bacterial elimination. *PGRP-SD*, which is induced to a greater degree in mated females, is best characterized as encoding a protein that recognizes Gram-positive bacterial infection (Bischoff *et al.* 2004; Wang *et al.* 2008). *IM4* is induced in response to bacterial infection and its transcription depends on the same signaling pathways that regulate antimicrobial peptide gene expression (Uttenweiler-Joseph *et al.* 1998), but the function of IM4 protein is unknown. *sphinx2* is a serine protease homolog and a paralog of *sphinx1*. Toll immune signaling is strongly reduced when both *sphinx1* and *sphinx2* are simultaneously knocked down using RNAi, but it is not yet clear whether *sphinx2* has an effect on immunity independent of *sphinx1* (Kambris *et al.* 2006). *r2d2* is part of the RNA interference machinery of *Drosophila* and plays an important role in antiviral immunity but not antibacterial immunity, and given that we performed infections with a bacterial pathogen, the implications of this result are unclear (Wang *et al.* 2006). *Gr28b* is involved in immune defense (Ayres *et al.* 2008) likely due to its role in regulating feeding behavior, which also alters defense against certain bacterial pathogens (Ayres and Schneider 2009).

The GO category “ATP biosynthetic process” contained genes encoding proteins with multiple roles in basic metabolic processes, such as ATP synthesis (*ATPsyn-gamma*) and ion transport (*Ca-P60A*, *ATP7*) (Table 3, Table S3), suggesting that basic metabolic functions may be differentially affected by infection depending on mating status.

**Figure 4:**
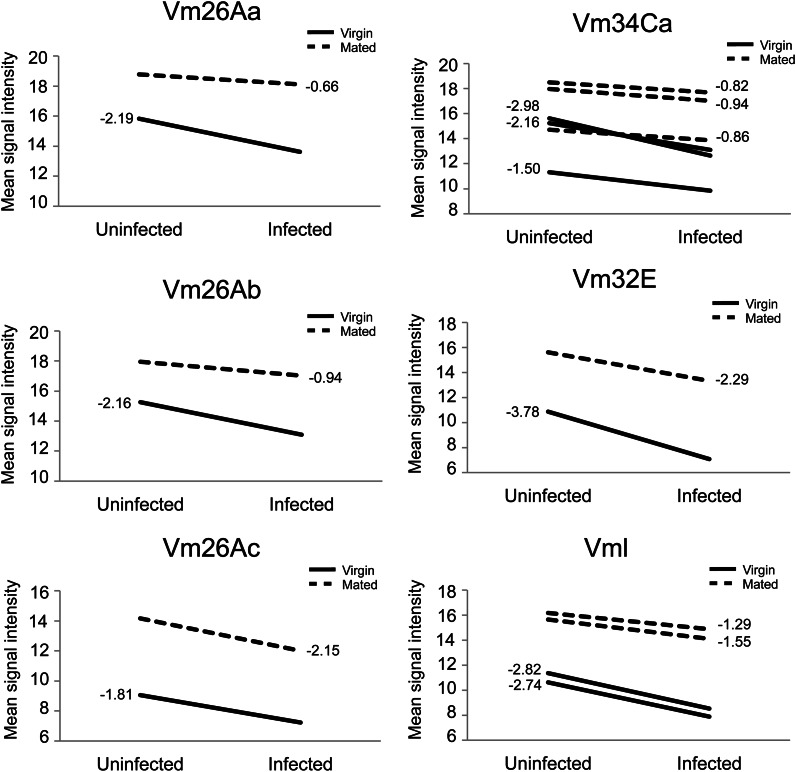
Vitelline membrane transcript abundances decrease after infection in egg-producing females. For all probesets that mapped to vitelline membrane genes, we determined averaged normalized signal intensity across all three biological replicates for each treatment. Only a single probeset exists on the array for Vm26Aa, Vm26Ab, Vm26Ac, and Vm32E, but Vm34Ca has three probesets and Vml has two. We then determined the change in mean signal intensity due to infection for virgin and mated females. These values are plotted to the left of each virgin line (solid) and to the right of each mated line (dashed) for each gene.

Our list of genes showing differential expression in virgin *vs.* mated females after infection also included a number of vitelline membrane formation genes: *Vm26Aa*, *Vm26Ab*, *Vm34Ca*, *Vm32E*, *Vml*, and *closca* (Table 3, Table S3). Of these, *Vml*, *Vm26Ab*, and *Vm34Ca* all exhibited nominally significant expression changes (uncorrected *P* < 0.05, Table S3). For all six vitelline membrane genes (the five above plus *Vm26Ac*), transcript abundance was greater in mated females compared to virgins, which is expected given that mated females actively produce higher numbers of eggs (Figure 4). We also found that, for all six genes, transcript abundance was reduced in response to infection in both mated and virgin females, which is consistent with a physiological trade-off between immune defense and reproduction (Figure 4). This reduction was more extreme in virgin females than in mated females in five out of six genes (Figure 4), which suggests that virgin females may improve their immune defense by withdrawing resources that would otherwise be spent on reproduction, whereas mated females may not have that option.

Vitelline membrane proteins are secreted during stages 8-10 of oogenesis by somatic follicle cells that surround the oocyte (Burke *et al.* 1987; Gigliotti *et al.* 1989). They form the vitelline membrane, the innermost layer of the *Drosophila* eggshell (Margaritis *et al.* 1980). The decrease in transcript abundance was more pronounced in virgin females relative to mated females. It is tempting to speculate that virgins may slow or alter oocyte progression when infected in a way that improves their ability to fight infection, and that it may be maladaptive or physiologically impossible for mated females to do the same. This infection-induced reduction in vitelline membrane transcripts could be the indirect result of a reallocation of resources toward immune defense and away from reproduction, or it may be the result of antagonistic signaling between the immune system and egg production. However, the nature of any interaction between vitelline membrane gene expression and immune defense, whether direct or indirect, will require further investigation.

We also identified genes that exhibited different magnitudes of expression change in response to infection in virgins *vs.* mated females using females that lack germlines. This contrast was measured as the absolute value of (comparison A – comparison B) being greater than 1.0. We found only 32 genes that met this basic criterion (Table S4). Of these 32, only six genes showed a nominally significant difference in expression between virgin and mated eggless females (uncorrected *P* < 0.05, Table S4). Notably, three genes from the list of 32 were also significant in this same comparison in egg-producing females (*takeout*, CG31775, CG32971). *Takeout* (*to*) shows sequence similarity with *Manduca sexta* juvenile hormone binding protein and has been hypothesized to act as a carrier of juvenile hormone (Sarov-Blat *et al.* 2000; Meunier *et al.* 2007). *to* is also implicated in the regulation of feeding behavior (Sarov-Blat *et al.* 2000). *to* expression is down-regulated in response to infection more strongly in virgins relative to mated females in both egg-producing and eggless females. Feeding behavior has the potential to affect immune defense (Ayres and Schneider 2009), but egg-producing, mated females are likely to have greater nutritional requirements than virgins. In general, the fact that eggless and egg-producing females share so few genes that change expression suggests that most of the differences we observed in egg-producing females (Table 3, Table S3) may in some way be contingent on the presence of a germline.

### The effect of infection status on expression of mating-responsive genes

Given that mated females suffer reduced systemic immune defense relative to virgins (Fedorka *et al.* 2007; Short and Lazzaro 2010), we were interested in identifying changes in gene expression that occur with mating in uninfected (comparison C in Figure 1, Table S1) and/or infected females (comparison D in Figure 1, Table S1). In several microarray studies, authors have investigated the differences in transcript abundance due to mating in females outside the context of infection (*e.g.*, Lawniczak and Begun 2004; McGraw *et al.* 2004,^2008^; Innocenti and Morrow 2009). These studies have reported up-regulation of a small number of immunity genes in response to mating, including increases in baseline expression of antimicrobial peptide genes that could potentially confer increased protection against infection. This result is seemingly in conflict with the observation that mated females perform more poorly than virgins in response to systemic bacterial infection (Fedorka *et al.* 2007; Short and Lazzaro 2010). However, all females used in these previous studies were uninfected. We specifically measured mating-induced changes in infected flies in addition to uninfected flies because we hypothesized that an ongoing infection may alter the female’s capacity to initiate her reproductive program.

**Figure 5:**
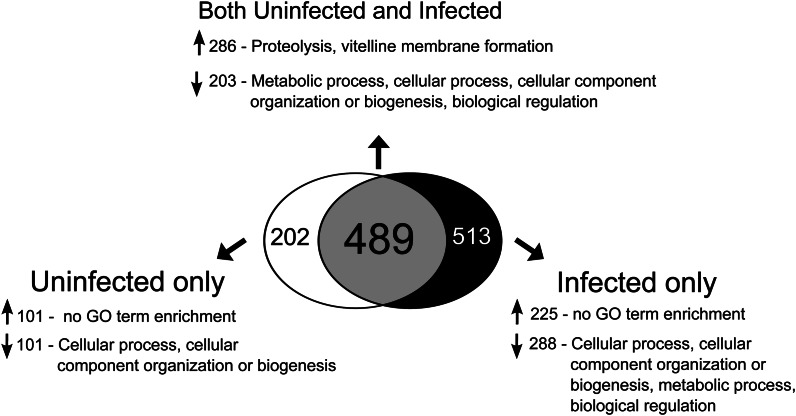
The effect of mating on transcript abundance in uninfected and infected females. We assayed for genes that showed significant twofold or greater differences in transcript abundance in virgin uninfected *vs.* mated uninfected treatments and in virgin infected *vs.* mated infected treatments. We then determined which genes change significantly in transcript abundance due to mating in both uninfected and infected females, only in uninfected, or only in infected females. GO term enrichment was determined for each set of genes using GOrilla, and REVIGO was used to reduce lists of GO terms to those least redundant. Upward-pointing arrows indicate genes with increased expression and downward-pointing arrows indicate genes with depressed expression. A Benjamini-Hochberg correction (Benjamini and Hochberg 1995) was performed to correct for multiple tests, and only GO terms that were significant after controlling for a false discovery rate of 5% were retained.

In our study, females were assayed at 12.5 hr after mating cessation for expression of genes significantly altered by mating in one or both infection states (comparison C and/or D in Figure 1, Figure 5, Table S1). There were 489 genes whose expression was altered by mating in both uninfected (comparison C in Figure 1) and infected females (comparison D in Figure 1, Table S1). Of these, 286 genes were significantly up-regulated in both uninfected and infected females and 203 genes were significantly down-regulated in both treatments (Figure 5, Table S1). A large number of genes were specifically altered in either uninfected or infected females. There were 101 genes significantly up-regulated and 101 genes significantly down-regulated after mating in uninfected females, but mating did not significantly alter the expression of these 202 genes in infected females (Figure 5, Table S1). Reciprocally, there were 225 genes that were up-regulated and 288 genes down-regulated in response to mating in infected females only (Figure 5, Table S1).

We tested for enrichment of GO terms among the genes whose expression was significantly altered by mating in the uninfected and/or infected females (Table 4). Among the genes with increased expression in both uninfected and infected females, we found enrichment of transcripts that function in proteolysis and formation of the vitelline membrane. We compared our results to those of McGraw *et al.* (2008), who assayed mating-induced changes in gene expression in uninfected females at approximately 12 hr after mating. This is similar to our time-point of approximately 12.5 hr post-mating. In McGraw *et al.* (2008), only 13 genes were found to change twofold or more after mating. Similar to our results (Table S1), McGraw *et al*. (2008) reported increased expression in genes involved in vitelline membrane formation (*Vm26Aa*, *Vm26Ab*, and *Vm34Ca*). They also reported increased expression in yolk protein genes (*Yp1* and *Yp2*) and an odorant binding protein (*Obp99a*), which we found to be significantly affected by mating in our study as well (Table S1).

**Table 4:** Biological process information for genes significantly altered by mating in uninfected and/or infected egg-producing females

Gene List	GO Term	GO Term Description	Corrected *P* Value	No. Genes in GO Category
Up significantly after mating in both uninfected and infected females	GO:0006508	Proteolysis	2.41E-13	51
	GO:0007305	Vitelline membrane formation involved in chorion-containing eggshell formation	4.93E-08	8
	GO:0043062	Extracellular structure organization	6.61E-05	9
	GO:0022412	Cellular process involved in reproduction in multicellular organism	5.62E-06	10
	GO:0010927	Cellular component assembly involved in morphogenesis	1.92E-03	10
Down significantly after mating in both uninfected and infected females	GO:0006259	DNA metabolic process	7.71E-14	28
	GO:0007051	Spindle organization	3.15E-08	21
	GO:0006996	Organelle organization	1.30E-06	42
	GO:0051276	Chromosome organization	1.70E-06	21
	GO:0007059	Chromosome segregation	1.92E-06	13
	GO:0090304	Nucleic acid metabolic process	4.76E-05	34
	GO:0006260	DNA replication	4.89E-05	10
	GO:0010564	Regulation of cell-cycle process	4.92E-05	15
	GO:0006139	Nucleobase-containing compound metabolic process	5.02E-05	39
	GO:0007010	Cytoskeleton organization	5.15E-05	25
	GO:0007017	Microtubule-based process	8.83E-05	23
	GO:0051726	Regulation of cell cycle	1.81E-04	17
	GO:0006270	DNA-dependent DNA replication initiation	1.92E-04	5
	GO:0034641	Cellular nitrogen compound metabolic process	1.92E-04	41
	GO:0006310	DNA recombination	6.89E-04	7
	GO:0051382	Kinetochore assembly	8.62E-04	3
	GO:0009132	Nucleoside diphosphate metabolic process	1.41E-03	4
	GO:0009220	Pyrimidine ribonucleotide biosynthetic process	1.51E-03	4
	GO:0070925	Organelle assembly	1.67E-03	7
	GO:0006807	Nitrogen compound metabolic process	1.96E-03	41
	GO:0009949	Polarity specification of anterior/posterior axis	5.18E-03	3
	GO:0071840	Cellular component organization or biogenesis	5.19E-03	46
	GO:0051313	Attachment of spindle microtubules to chromosome	5.32E-03	3
	GO:0006165	Nucleoside diphosphate phosphorylation	5.77E-03	3
	GO:0051383	Kinetochore organization	8.49E-03	3
	GO:0065003	Macromolecular complex assembly	8.56E-03	11
	GO:0065001	Specification of axis polarity	8.68E-03	3
	GO:0006333	Chromatin assembly or disassembly	8.68E-03	5
	GO:0051303	Establishment of chromosome localization	1.20E-02	4
	GO:0046939	Nucleotide phosphorylation	1.85E-02	3
	GO:0072527	Pyrimidine-containing compound metabolic process	2.09E-02	4
	GO:0006974	Response to DNA damage stimulus	2.28E-02	12
	GO:0045035	Sensory organ precursor cell division	2.31E-02	3
	GO:0000910	Cytokinesis	2.33E-02	7
	GO:0033043	Regulation of organelle organization	2.48E-02	9
	GO:0001709	Cell fate determination	3.10E-02	8
	GO:0043933	Macromolecular complex subunit organization	3.12E-02	11
	GO:0009994	Oocyte differentiation	3.13E-02	3
	GO:0044260	Cellular macromolecule metabolic process	3.32E-02	47
	GO:0001964	Startle response	4.86E-02	3
Up significantly after mating in only uninfected females	No enrichment			
Down significantly after mating in only uninfected females	GO:0051276	Chromosome organization	6.78E-11	20
	GO:0006325	Chromatin organization	1.61E-10	16
	GO:0034728	Nucleosome organization	1.37E-07	9
	GO:0065004	Protein−DNA complex assembly	2.24E-07	9
	GO:0071824	Protein−DNA complex subunit organization	3.61E-07	9
	GO:0006996	Organelle organization	5.52E-06	26
	GO:0043933	Macromolecular complex subunit organization	3.05E-05	12
	GO:0071840	Cellular component organization or biogenesis	4.71E-04	29
	GO:0007059	Chromosome segregation	2.06E-03	7
	GO:0048869	Cellular developmental process	9.17E-03	22
	GO:0006259	DNA metabolic process	1.60E-02	9
	GO:0071844	Cellular component assembly at cellular level	1.62E-02	12
	GO:0007049	Cell cycle	2.16E-02	6
	GO:0051726	Regulation of cell cycle	2.99E-02	9
	GO:0000082	G1/S transition of mitotic cell cycle	3.05E-02	3
	GO:0051310	Metaphase plate congression	3.65E-02	3
	GO:0030154	Cell differentiation	3.73E-02	15
Up significantly after mating in ONLY Infected females	No enrichment			
Down significantly after mating in only infected females	GO:0051276	Chromosome organization	1.83E-04	22
	GO:0007346	Regulation of mitotic cell cycle	4.29E-04	17
	GO:0051726	Regulation of cell cycle	6.68E-04	20
	GO:0006259	DNA metabolic process	1.68E-03	18
	GO:0045596	Negative regulation of cell differentiation	6.94E-03	11
	GO:0050794	Regulation of cellular process	8.05E-03	78
	GO:0006281	DNA repair	8.25E-03	10
	GO:0009794	Regulation of mitotic cell cycle, embryonic	8.78E-03	4
	GO:0065007	Biological regulation	9.06E-03	88
	GO:0050789	Regulation of biological process	1.01E-02	82
	GO:0007059	Chromosome segregation	1.05E-02	10
	GO:0044260	Cellular macromolecule metabolic process	1.09E-02	64
	GO:0006996	Organelle organization	1.18E-02	40
	GO:0010468	Regulation of gene expression	1.64E-02	38
	GO:0019222	Regulation of metabolic process	1.90E-02	45
	GO:0032880	Regulation of protein localization	2.15E-02	7
	GO:0051093	Negative regulation of developmental process	2.22E-02	11
	GO:0048519	Negative regulation of biological process	2.51E-02	31
	GO:0050793	Regulation of developmental process	3.48E-02	21
	GO:0006464	Protein modification process	3.99E-02	28
	GO:0048523	Negative regulation of cellular process	4.10E-02	27
	GO:0045132	Meiotic chromosome segregation	4.14E-02	6
	GO:0043412	Macromolecule modification	4.16E-02	29
	GO:0051017	Actin filament bundle assembly	4.38E-02	4
	GO:0042683	Negative regulation of compound eye cone cell fate specification	4.40E-02	2
	GO:0051301	Cell division	4.41E-02	9
	GO:0006325	Chromatin organization	4.42E-02	11
	GO:0043161	Proteasomal ubiquitin-dependent protein catabolic process	4.53E-02	4
	GO:0006348	Chromatin silencing at telomere	4.58E-02	2
	GO:0090068	Positive regulation of cell cycle process	4.64E-02	4
	GO:0045995	Regulation of embryonic development	4.72E-02	7
	GO:0071840	Cellular component organization or biogenesis	4.89E-02	53

The post-mating increases in vitelline membrane gene expression we found are expected given that vitelline membrane genes are highly expressed during the vitellogenic stages of oogenesis (stages 8−10; Burke *et al.* 1987; Gigliotti *et al.* 1989), and mated females are actively producing high numbers of vitellogenic oocytes at approximately12 hr post-mating when these measurements were taken (Heifetz *et al.* 2001).

Genes encoding proteolysis regulators could be involved in many possible post-mating functions, including the processing of seminal fluid proteins (*e.g.*, Pilpel *et al.* 2008). Proteolysis-regulator encoding genes also function in immunity, and act to regulate melanization and humoral immune signaling (Cerenius and Söderhäll 2004; Wang and Ligoxygakis 2006). Many of the proteolysis genes we detected as being up-regulated by mating belong to the *Jonah* gene family (*Jon65Aii*, *Jon65Aiii*, *Jon65Aiv*, *Jon25Bi*, *Jon25Bii*, *Jon99Cii*, *Jon44E*, *Jon74E*, *Jon99Fi*, *Jon99Fii*, and *Jon66Ci*, Table S1). *Jonah* genes have previously been reported to be expressed only in the midgut (Akam and Carlson 1985). *Jonah* genes are down-regulated in response to infection (this study: *Jon99Fi* and *Jon99Ci*, Table S1; De Gregorio *et al.* 2001: *Jon44E*, *Jon25Bi*, *Jon25Bii*, *Jon99Fi*). The induction of *Jonah* genes by mating and their repression by infection may indicate one potential antagonistic pleiotropy between immunity and reproduction, perhaps mediated by differences in feeding behavior or nutritional uptake.

Genes with reduced transcript abundance after mating in both uninfected and infected females were enriched for many GO terms involved in cellular replication, including chromosome segregation, regulation of cell cycle, DNA replication, and spindle organization (Table 4). These and other related GO categories were also enriched among genes whose expression is repressed by mating specifically in uninfected females or specifically in infected females. It initially surprised us that these transcripts were reduced in abundance, given that oocyte production, which increases after mating, requires cell division and reorganization. However, mated females lay a large number of mature eggs shortly after mating, and because of this have fewer late-stage oocytes (stages 13−14) than virgins at the time of our assay (Heifetz *et al.* 2001). We hypothesized that many of these transcripts may actually be maternally deposited into late-stage oocytes and the apparent reduction in the transcript level of these genes may merely reflect the fact that the late-stage oocytes bearing these transcripts have already begun to be laid by mated females. To test this, we compared our list of down-regulated genes to two independently generated lists of maternal transcripts (Hooper *et al.* 2007; Tomancak *et al.* 2002, 2007) and found that 62.1% of the genes reduced due to mating in both uninfected and infected females have been identified as being maternally deposited into oocytes. Similarly, 61.5% of the genes whose transcript abundance was significantly reduced only in infected females and 42.6% of those reduced only in uninfected females are maternally deposited. Although this does not account for all of the genes showing reduced expression after mating in uninfected and/or infected females, we think that maternal deposition of transcripts into oocytes probably accounts for much of the observed result.

Although uninfected and infected females demonstrated generally similar patterns of change in transcript abundance after mating (Table 4), we note that the GO term “humoral immune response” (GO:0006959) was enriched among genes that showed increased transcript abundance after mating specifically in infected females, but it did not survive correction for multiple testing (*P* = 6.36 × 10^−5^, corrected *P* = 0.102, data not shown). Because our multiple testing correction was rather stringent, we thought that this result warranted further investigation. This GO term included two immune-induced molecules (*IM4* and *IM10*) and five genes with lysozyme activity (*LysB*, *LysC*, *LysD*, *LysE*, and *CG16799*) whose expression was significantly greater after mating in infected females but not in uninfected females (Table S1). The lysozyme genes up-regulated in response to mating comprise the *LysD*-like gene family, which is thought to be expressed only in the gut of adult flies (Daffre *et al.* 1994). It is possible that this result is related to infection-induced changes in the gut rather than being a direct result of systemic infection. Mating has been shown to increase food intake (Carvalho *et al.* 2006), and these gut-specific mating-induced changes in gene expression may be a result of altered feeding behavior.

**Figure 6:**
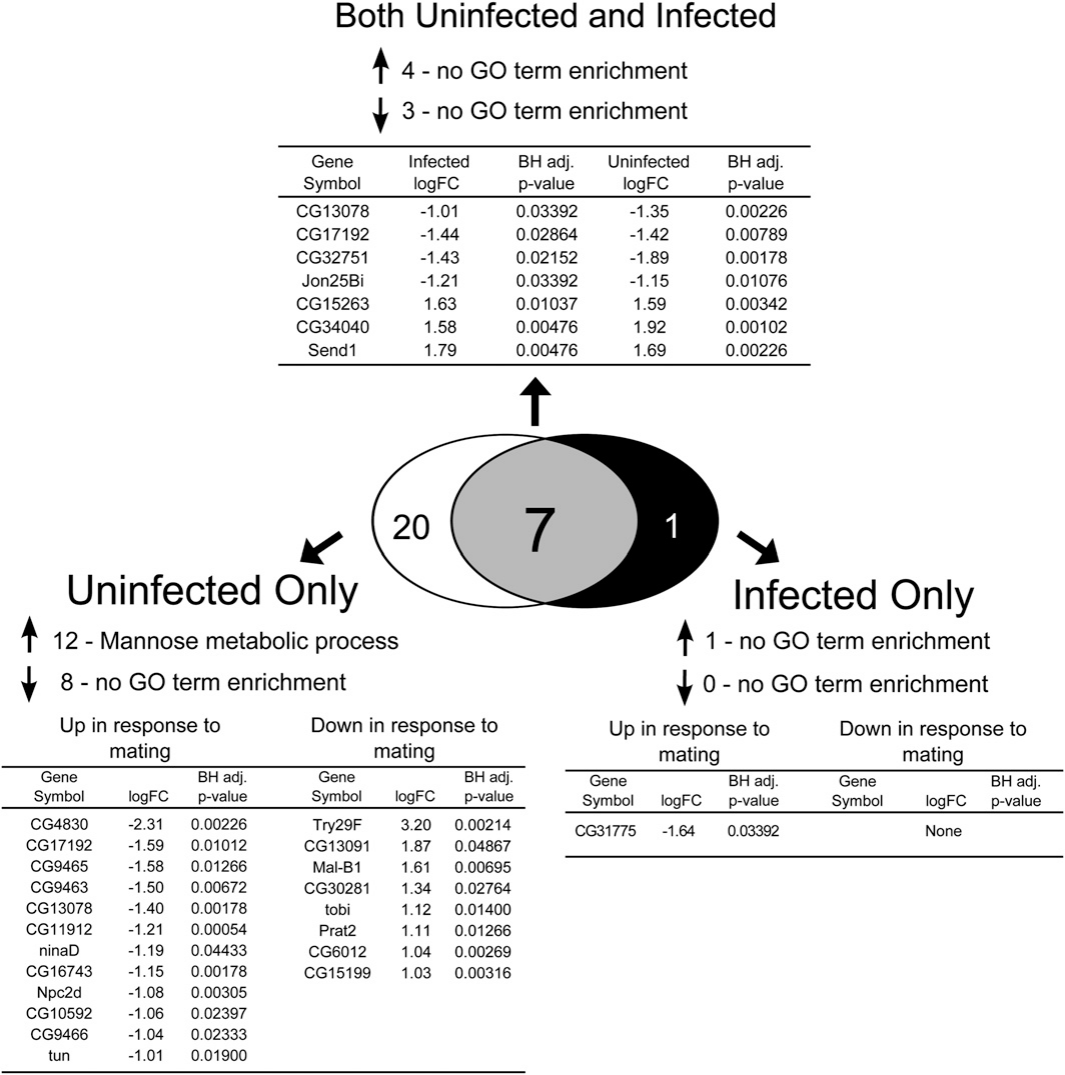
The effect of mating on transcript abundance in uninfected and infected eggless females. We assayed for genes that showed significant twofold or greater differences in expression in virgin uninfected *vs.* mated uninfected treatments and in virgin infected *vs.* mated infected treatments. We then determined which genes have significantly altered expression due to mating in both uninfected and infected females, only in uninfected females, or only in infected females. Fold change values are in log_2_ units and are expressed as virgin minus mated signal; therefore, a negative logFC represents increased expression in response to mating whereas a positive logFC represents decreased expression in response to mating. In instances in which more than one probe indicated a significant change in expression for a particular gene, the probeset with the largest fold change is listed. GO term enrichment was determined using GOrilla. Upward-pointing arrows indicate genes with increased expression and downward-pointing arrows indicate genes with depressed expression. A Benjamini-Hochberg correction (Benjamini and Hochberg 1995) was performed to correct for multiple tests, and only GO terms that were significant after controlling for a false-discovery rate of 5% were retained.

In eggless females, mating itself induced very few transcriptional changes. Only seven genes were altered after mating in both uninfected and infected eggless females (Figure 6). One of these genes was *Jon25Bi*, suggesting that the post-mating change in transcription of *Jonah* genes by egg-producing females is at least partly independent of the presence of a germline. Uninfected females exhibited increases in transcript abundance of genes enriched for mannose metabolism after mating, a result that was not observed in infected females after mating (GO term *P* = 7.52 × 10^−4^; Figure 6). It is possible that this may be indicative of germline-independent mating-induced changes in metabolism that fail to occur when the female is infected, though more data are needed to develop this interpretation beyond speculation.

In this work, we identified changes in gene expression that occur in response to mating and infection with the goal of gaining a better understanding of the molecular mechanisms that contribute to post-mating immunosuppression in female *D. melanogaster*. We found several immune-related genes to be differentially affected by infection in virgin compared to mated females. These included opsonizing factors, antimicrobial peptides, and genes in the *Turandot* family, indicating multiple aspects of immune system activity that could potentially contribute to the reduced ability of mated females to resist and survive bacterial infection. We also found that females reduce expression of genes involved in vitelline membrane and chorion production upon infection, and that this effect is more pronounced in virgins than in mated females. This finding suggests that females may reduce investment in egg production to fight infection and that variation in immune defense may be in part dependent on the ability of females to alter their current reproductive output. Finally, we note that the expression of genes involved in feeding behavior (*takeout* and *Gr28b*) was differentially regulated after infection in virgins compared to mated females. We also found that a number of gut-specific genes were affected by mating status but in an infection-status specific manner (*Jon99Ci*, *LysD-like* genes). Although the full implications of these results remain to be explored, the initial observations suggest that the differences we see in immune defense between virgins and mated females may stem partially from differential changes in feeding behavior after mating and infection.

## Supplementary Material

Supporting Information
