# Supplementary material for: Reproductive Status Alters Transcriptomic Response to Infection in Female Drosophila melanogaster
Source: G3 (Bethesda). 2013 May 1;3(5):827–40. doi: 10.1534/g3.112.005306 (PMC3656730; doi:10.1534/g3.112.005306)
Supplement: Supporting Information [file supp_3_5_827__index.html]

Reproductive Status Alters Transcriptomic Response to Infection in Female Drosophila melanogaster — Reproductive Status Alters Transcriptomic Response to Infection in Female Drosophila melanogaster — Supporting Information 

# Reproductive Status Alters Transcriptomic Response to Infection in Female *Drosophila melanogaster*

## Supporting Information for Short and Lazzaro, 2013

**Files in this Data Supplement:**

- Supporting Information - Figures S1-S3, File S1, and Tables S1-S7 (PDF, 157 KB)
- Figure S1 - The effect of mating status on bacterial load at multiple time points post-infection (PDF, 78 KB)
- Figure S2 - Log2 fold-change values for egg-producing females from the microarray experiment versus those from qRT-PCR validation (PDF, 77 KB)
- Figure S3 - Log2 fold-change values for eggless females from the microarray experiment versus those from qRT-PCR validation (PDF, 77 KB)
- File S1 - MICROARRAY VALIDATION BY QPCR (PDF, 98 KB)
- Table S1 - Log2 fold change values and Benjamini-Hochberg adjusted P-values for all treatment comparisons and all probesets for egg-producing females (.csv, 2.9 MB)
- Table S2 - Log2 fold change values and Benjamini-Hochberg adjusted P-values for all treatment comparisons and all probesets for germline-less females (.csv, 2.9 MB)
- Table S3 - Genes for which the infection response of virgin egg-producing females differs from that of mated egg-producing females by at least two-fold (comparison A minus comparison B from Figure 1) (.xlsx, 64 KB)
- Table S4 - Genes for which the infection response of virgin germline-less females differs from that of mated germline-less females by at least two-fold (Comparison A minus Comparison B from Figure 1) (.xlsx, 17 KB)
- Table S5 - qPCR Primer sequences used for microarray validation (.xlsx, 12 KB)
- Table S6 - qPCR validation of microarray log2 fold-changes for nine genes for egg-producing females (.xlsx, 15 KB)
- Table S7 - qPCR validation of microarray log2 fold-changes for nine genes for eggless females (.xlsx, 16 KB)
