# Supplementary material for: Reproductive Status Alters Transcriptomic Response to Infection in Female Drosophila melanogaster
Source: G3 (Bethesda). 2013 May 1;3(5):827–40. doi: 10.1534/g3.112.005306 (PMC3656730; doi:10.1534/g3.112.005306)
Supplement: Supporting Information [file supp_g3.112.005306_005306SI.pdf]

G3 · Genes | Genomes | Genetics

Reproductive status alters transcriptomic response to infection in female *Drosophila melanogaster*

Sarah M. Short[*,1] and Brian P. Lazzaro[*]

*Field of Genetics and Development; Cornell University; Ithaca, NY 14853

1. Present address: W. Harry Feinstone Department of Molecular Microbiology and Immunology; Bloomberg School of Public Health; Johns Hopkins University; Baltimore, MD 21205

DOI: 10.1534/g3.112.005306

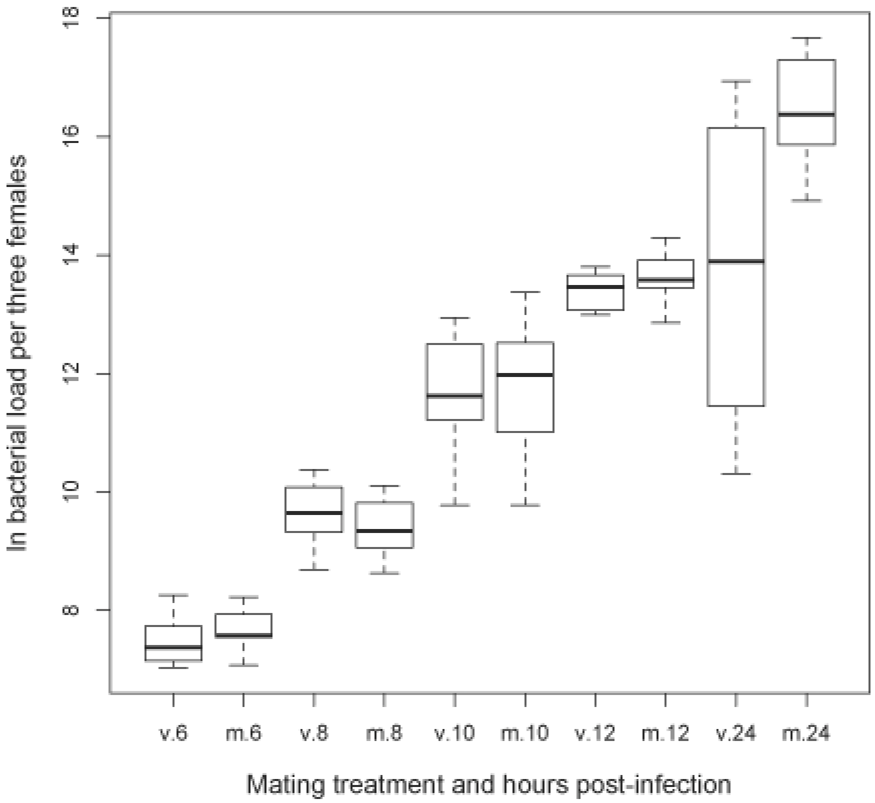

Figure S1 The effect of mating status on bacterial load at multiple time points post-infection. We infected mated females and virgin controls from the strain Canton S with the bacterium *P. rettgeri* at approximately 2.5 hours post-mating. At six, eight, ten, twelve and twenty-four hours post-infection, we assayed bacterial levels present in virgin and mated females. Females were homogenized in pools of three in sterile LB and an aliquot of homogenate was plated on LB agar plates using a spiral plater (Microbiology International). The number of bacterial colonies that grew from the aliquot was used to calculate the number of viable bacteria in each pool of three female flies.

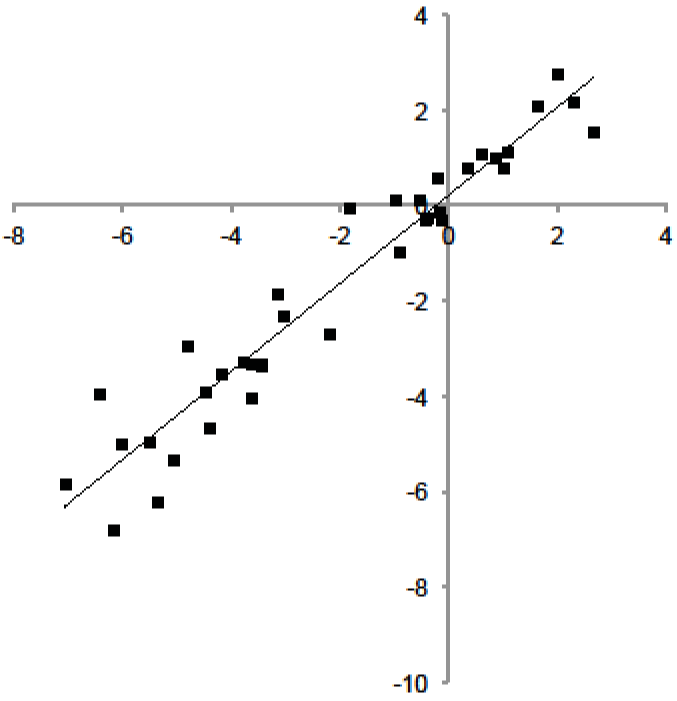

Figure S2 Log_2 fold-change values for egg-producing females from the microarray experiment versus those from qRT-PCR validation. We synthesized cDNA from the same RNA samples used for the microarray experiment and measured gene expression by qPCR for nine genes. We then estimated log_2 fold-change values for virgin uninfected vs. virgin infected, mated uninfected vs. mated infected, virgin uninfected vs. mated uninfected, and virgin infected vs. mated infected using a Tukey's test. We then plotted these log_2 fold-changes (x-axis) against the log_2 fold change values we obtained for these same genes from the microarray experiment (y-axis). All values plotted in this figure can be found in Table S6. For many of the genes we measured, there was more than one independent probeset on the microarray. In these cases, we picked one probeset at random to include in this figure, with the exception of *TotM* and *BobA*. For these two genes, one probeset showed very low mean transcript abundance for all treatments (*TotM:* ProbeUID 21055, *BobA:* ProbeUID 11736). Using BLAST (blast.ncbi.nlm.nih.gov), we found that both probes lack specificity for their target sequences and we therefore chose to exclude them from this analysis. The correlation between the microarray and qPCR data was very highly significant (Pearson's correlation coefficient $r = 0.96$, $p < 0.0001$).

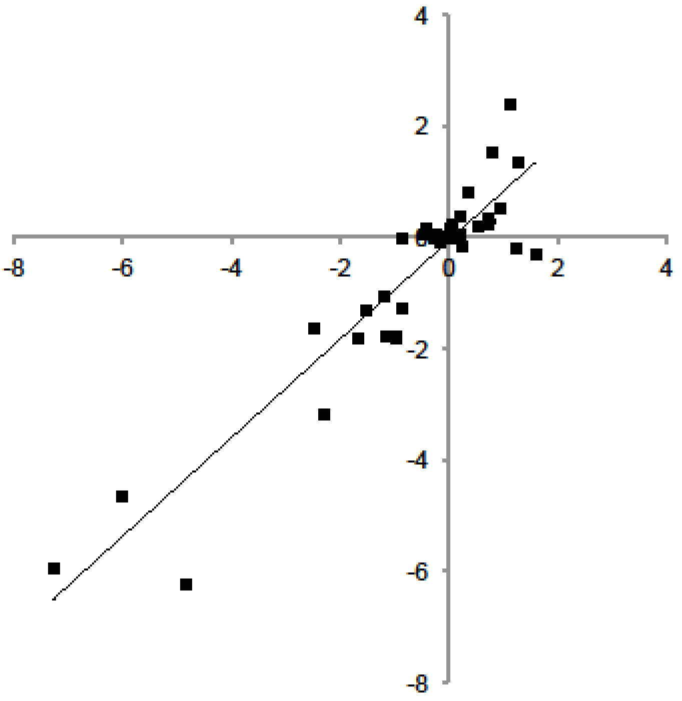

Figure S3 Log$_2$ fold-change values for eggless females from the microarray experiment versus those from qRT-PCR validation. We synthesized cDNA from the same samples used for the microarray experiment and measured gene expression by qPCR for nine genes. We then estimated log$_2$ fold-change values for virgin uninfected vs. virgin infected, mated uninfected vs. mated infected, virgin uninfected vs. mated uninfected, and virgin infected vs. mated infected using a Tukey's test. We then plotted these log$_2$ fold-changes (x-axis) against the log$_2$ fold change values we obtained for these same genes from the microarray experiment (y-axis). All values plotted in this figure can be found in Table S7. For many of the genes we measured, there was more than one independent probeset on the microarray. In these cases, we picked one probeset at random to include in this figure, with the exception of *TotM* and *BobA*. For these two genes, one probeset showed very low mean transcript abundance for all treatments (*TotM:* ProbeUID 21055, *BobA:* ProbeUID 11736). Using BLAST (blast.ncbi.nlm.nih.gov), we found that both probes lack specificity for their target sequences and we therefore chose to exclude them from this analysis. The correlation between the microarray and qPCR data was very highly significant (Pearson's correlation coefficient $r = 0.92$, $p < 0.0001$).

File S1

MICROARRAY VALIDATION BY QPCR

Sample prep and qRT-PCR: In order to validate the microarray hybridizations and analysis, we performed gene expression analysis using qRT-PCR on cDNA made from aliquots of the same RNA samples used for the microarrays. Residual genomic DNA was removed from the RNA samples using TURBO DNA-*free* (Ambion). We then synthesized cDNA from 1ug of DNAse-treated RNA using M-MLV reverse transcriptase (Promega) according to the manufacturer's instructions. We diluted the cDNA 1:5 and performed qRT-PCR using iQ SYBR Green Supermix (Bio-Rad) according to the manufacturer's instructions. Primer sequences used for each gene can be found in Table S5. We chose to validate eight genes for which the infection response of virgins was at least two-fold different from the infection response of mated females. All genes that met this criterion are found in Table S3. We sorted Table S3 by absolute log fold-change and then determined quartile ranges of the absolute log fold-change values. We chose one gene from the first quartile, three from second quartile, three from the third quartile and one from the fourth quartile. In addition to these eight genes, we also validated one additional gene from the *Turandot* family. Our intention was to validate genes that were biologically relevant to our study but that showed a broad range of treatment response values. We also measured expression of the housekeeping genes *αTub84B* and *Actin42A,* which we used as reference genes in our analysis. Measuring gene expression relative to expression of a housekeeping gene allowed us to control for differential extraction yield or other incidental differences across samples.

Data analysis and result summary: In order to determine the differences in gene expression across mating and infection statuses, we performed an analysis of variance for each gene using the following model: $Y_{ijk} = \mu +$ *αTub84B +* experimental replicate_i + mating status_j + infection status_k + mating $\text{status}_j \times$ infection $\text{status}_k + \varepsilon_{ijk}$, where Y was the critical threshold (C_T) value for all samples for the gene of interest, experimental replicate_i (i = 1,3) included the three biological replicates over which the samples were collected, mating status_j (j = 1,2) indicated whether the samples were virgin or mated females, and infection status_k (k = 1,2) represented whether the samples were infected or uninfected. Analyses for all genes were performed for egg-producing females and then repeated separately for eggless females. After performing an ANVOA, we then performed a Tukey's Honest Significant Difference test on the interaction term (mating $\text{status}_j \times$ infection status_k). This provided estimates of the differences in $\log_2$ gene expression between all comparisons of interest as well as p-values corrected for the multiple treatment comparisons. We repeated this entire analysis for all genes using *Actin42A* and found the results to be qualitatively similar to those obtained using *αTub84B* as a reference gene.

Log$_2$ fold-change values measured by qPCR for all genes for egg-producing females are listed in Table S6, as are the corresponding log$_2$ fold-change values given by the microarray experiment. These same data for eggless females are listed in Table S7. As a general estimate of the overall similarity between our microarray results and our qPCR validation, we plotted all qPCR log$_2$ fold-change values against the log$_2$ fold-change for one randomly-chosen probest for each gene from the microarray (egg-producing females: Figure S2, eggless females: Figure S3). We then calculated the Pearson correlation coefficient. We found a high degree of similarity between the microarray results and our qPCR validation for both egg-producing females ($r = 0.96$, $p < 0.0001$, Figure S2, Table S6) and eggless females ($r=0.92$, $p < 0.0001$, Figure S3, Table S7).

Supplemental references:

Ponton F., Chapuis M.-P., Pernice M., Sword G. A., Simpson S. J., 2011 Evaluation for potential reference genes for reverse transcription-qPCR studies of physiological responses in *Drosophila melanogaster. J. Ins. Physiol.* **57:** 840-50.

Rand M. D., Bland C. E., Bond J., 2008 Methylmercury activates enhancer-of-split and bearded complex genes independent of the notch receptor. *Toxicol. Sci.* **104:** 163-76.

Ye Y. H., Chenoweth S. F., McGraw E. A., 2009 Effective but costly, evolved mechanisms of defense against a virulent opportunistic pathogen in *Drosophila melanogaster. PLoS Pathog.* **5:** e1000385.

Zhang J., Marshall K. E., Westwood T., Clark M. S., Sinclair B. J., 2011 Divergent transcriptomic responses to repeated and single cold exposures in *Drosophila melanogaster. J. Exp. Biol.* **214:** 4021-9.

Tables S1-S7
Available for download at http://www.g3journal.org/lookup/suppl/doi:10.1534/g3.112.005306/-/DC1

Table S1 Log_2 fold change values and Benjamini-Hochberg adjusted P-values for all treatment comparisons and all probesets for egg-producing females. Highlighted cells are those that are significant at $p < 0.05$ and have a log_2 fold change of at least 1.0. Gene names from Agilent are provided, as are gene names that we verified manually for use in GO analysis.

Table S2 Log_2 fold change values and Benjamini-Hochberg adjusted P-values for all treatment comparisons and all probesets for germline-less females. Highlighted cells are those that are significant at $p < 0.05$ and have a log_2 fold change of at least 1.0. Gene names from Agilent are provided, as are gene names that we verified manually for use in GO analysis.

Table S3 Genes for which the infection response of virgin **egg-producing** females differs from that of mated **egg-producing** females by at least two-fold (comparison A minus comparison B from Figure 1). In addition to the log_2 difference between the infection responses of virgin and mated females, this table also contains the corresponding log_2 values for virgin and mated infection response for each probe.

Table S4 Genes for which the infection response of virgin **germline-less** females differs from that of mated **germline-less** females by at least two-fold (Comparison A minus Comparison B from Figure 1). In addition to the log_2 difference between Comparison A and Comparison B, this table also contains the corresponding Comparison A and Comparison B log_2 values for each probe.

Table S5 qPCR Primer sequences used for microarray validation

Table S6 qPCR validation of microarray log_2 fold-changes for nine genes for **egg-producing** females

Table S7 qPCR validation of microarray log_2 fold-changes for nine genes for **eggless** females
