# Supplementary material for: Reproductive Status Alters Transcriptomic Response to Infection in Female Drosophila melanogaster
Source: G3 (Bethesda). 2013 May 1;3(5):827–40. doi: 10.1534/g3.112.005306 (PMC3656730; doi:10.1534/g3.112.005306)
Supplement: Supporting Information [file supp_g3.112.005306_FigureS1.pdf]

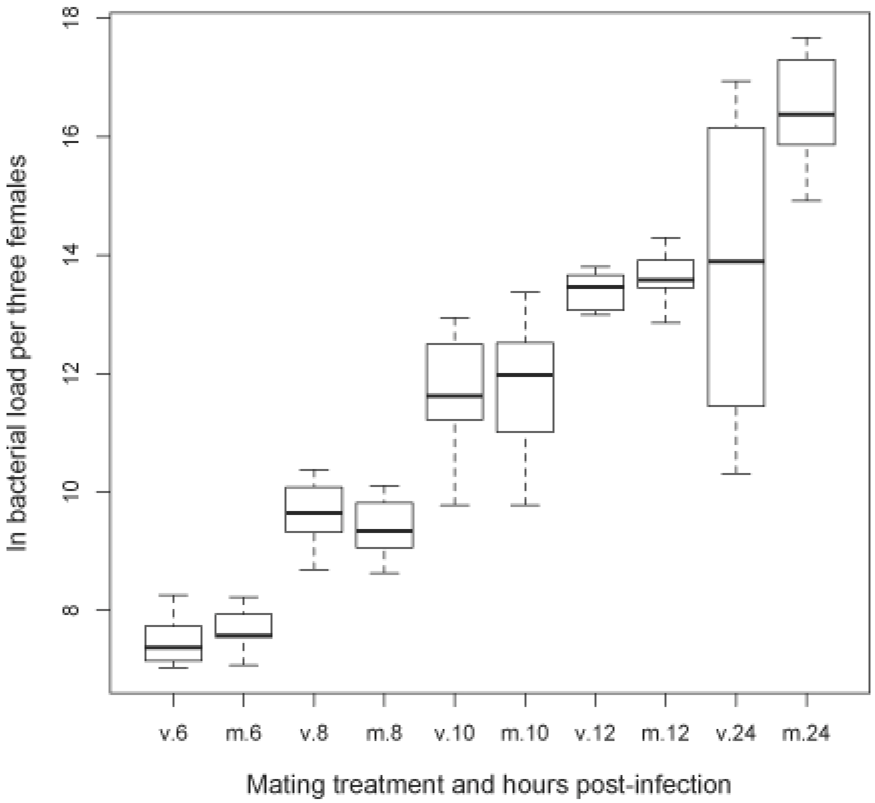

Figure S1 The effect of mating status on bacterial load at multiple time points post-infection. We infected mated females and virgin controls from the strain Canton S with the bacterium *P. rettgeri* at approximately 2.5 hours post-mating. At six, eight, ten, twelve and twenty-four hours post-infection, we assayed bacterial levels present in virgin and mated females. Females were homogenized in pools of three in sterile LB and an aliquot of homogenate was plated on LB agar plates using a spiral plater (Microbiology International). The number of bacterial colonies that grew from the aliquot was used to calculate the number of viable bacteria in each pool of three female flies.
