# Supplementary material for: Reproductive Status Alters Transcriptomic Response to Infection in Female Drosophila melanogaster
Source: G3 (Bethesda). 2013 May 1;3(5):827–40. doi: 10.1534/g3.112.005306 (PMC3656730; doi:10.1534/g3.112.005306)
Supplement: Supporting Information [file supp_g3.112.005306_FigureS2.pdf]

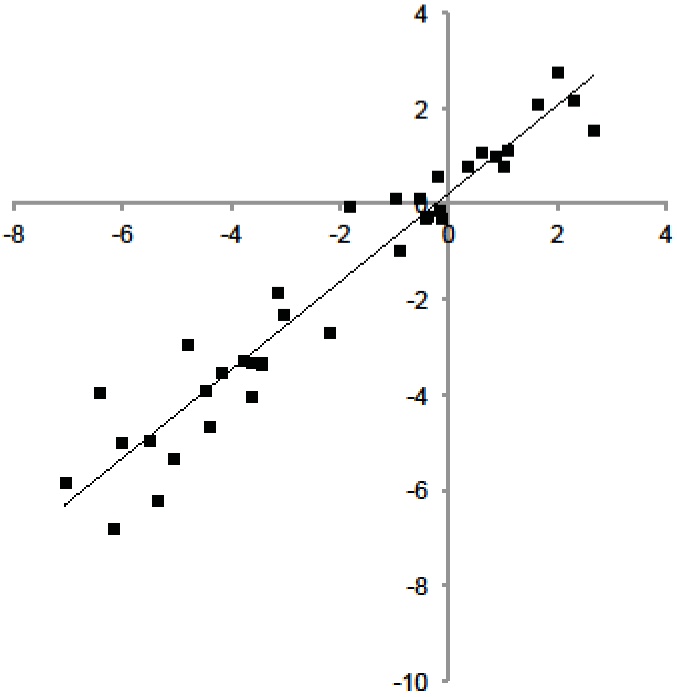

Figure S2 Log$_2$ fold-change values for egg-producing females from the microarray experiment versus those from qRT-PCR validation. We synthesized cDNA from the same RNA samples used for the microarray experiment and measured gene expression by qPCR for nine genes. We then estimated log$_2$ fold-change values for virgin uninfected vs. virgin infected, mated uninfected vs. mated infected, virgin uninfected vs. mated uninfected, and virgin infected vs. mated infected using a Tukey's test. We then plotted these log$_2$ fold-changes (x-axis) against the log$_2$ fold change values we obtained for these same genes from the microarray experiment (y-axis). All values plotted in this figure can be found in Table S6. For many of the genes we measured, there was more than one independent probeset on the microarray. In these cases, we picked one probeset at random to include in this figure, with the exception of *TotM* and *BobA*. For these two genes, one probeset showed very low mean transcript abundance for all treatments (*TotM:* ProbeUID 21055, *BobA:* ProbeUID 11736). Using BLAST (blast.ncbi.nlm.nih.gov), we found that both probes lack specificity for their target sequences and we therefore chose to exclude them from this analysis. The correlation between the microarray and qPCR data was very highly significant (Pearson's correlation coefficient r = 0.96, $p <$ 0.0001).
