# Supplementary material for: Reproductive Status Alters Transcriptomic Response to Infection in Female Drosophila melanogaster
Source: G3 (Bethesda). 2013 May 1;3(5):827–40. doi: 10.1534/g3.112.005306 (PMC3656730; doi:10.1534/g3.112.005306)
Supplement: Supporting Information [file supp_g3.112.005306_FileS1.pdf]

File S1

MICROARRAY VALIDATION BY QPCR

Sample prep and qRT-PCR: In order to validate the microarray hybridizations and analysis, we performed gene expression analysis using qRT-PCR on cDNA made from aliquots of the same RNA samples used for the microarrays. Residual genomic DNA was removed from the RNA samples using TURBO DNA-*free* (Ambion). We then synthesized cDNA from 1ug of DNAse-treated RNA using M-MLV reverse transcriptase (Promega) according to the manufacturer's instructions. We diluted the cDNA 1:5 and performed qRT-PCR using iQ SYBR Green Supermix (Bio-Rad) according to the manufacturer's instructions. Primer sequences used for each gene can be found in Table S5. We chose to validate eight genes for which the infection response of virgins was at least two-fold different from the infection response of mated females. All genes that met this criterion are found in Table S3. We sorted Table S3 by absolute log fold-change and then determined quartile ranges of the absolute log fold-change values. We chose one gene from the first quartile, three from second quartile, three from the third quartile and one from the fourth quartile. In addition to these eight genes, we also validated one additional gene from the *Turandot* family. Our intention was to validate genes that were biologically relevant to our study but that showed a broad range of treatment response values. We also measured expression of the housekeeping genes *αTub84B* and *Actin42A,* which we used as reference genes in our analysis. Measuring gene expression relative to expression of a housekeeping gene allowed us to control for differential extraction yield or other incidental differences across samples.

Data analysis and result summary: In order to determine the differences in gene expression across mating and infection statuses, we performed an analysis of variance for each gene using the following model: $Y_{ijk} = \mu +$ *αTub84B +* experimental replicate$_i$ + mating status$_j$ + infection status$_k$ + mating status$_j$ × infection status$_k$ + ε_{ijk}, where Y was the critical threshold (C_T) value for all samples for the gene of interest, experimental replicate$_i$ (i = 1,3) included the three biological replicates over which the samples were collected, mating status$_j$ (j = 1,2) indicated whether the samples were virgin or mated females, and infection status$_k$ (k = 1,2) represented whether the samples were infected or uninfected. Analyses for all genes were performed for egg-producing females and then repeated separately for eggless females. After performing an ANVOA, we then performed a Tukey's Honest Significant Difference test on the interaction term (mating status$_j$ × infection status$_k$). This provided estimates of the differences in $\log_2$ gene expression between all comparisons of interest as well as p-values corrected for the multiple treatment comparisons. We repeated this entire analysis for all genes using *Actin42A* and found the results to be qualitatively similar to those obtained using *αTub84B* as a reference gene.

Log_2 fold-change values measured by qPCR for all genes for egg-producing females are listed in Table S6, as are the corresponding log_2 fold-change values given by the microarray experiment. These same data for eggless females are listed in Table S7. As a general estimate of the overall similarity between our microarray results and our qPCR validation, we plotted all qPCR log_2 fold-change values against the log_2 fold-change for one randomly-chosen probest for each gene from the microarray (egg-producing females: Figure S2, eggless females: Figure S3). We then calculated the Pearson correlation coefficient. We found a high degree of similarity between the microarray results and our qPCR validation for both egg-producing females ($r = 0.96$, $p < 0.0001$, Figure S2, Table S6) and eggless females ($r=0.92$, $p < 0.0001$, Figure S3, Table S7).

Supplemental references:

Ponton F., Chapuis M.-P., Pernice M., Sword G. A., Simpson S. J., 2011 Evaluation for potential reference genes for reverse transcription-qPCR studies of physiological responses in *Drosophila melanogaster. J. Ins. Physiol.* **57:** 840-50.

Rand M. D., Bland C. E., Bond J., 2008 Methylmercury activates enhancer-of-split and bearded complex genes independent of the notch receptor. *Toxicol. Sci.* **104:** 163-76.

Ye Y. H., Chenoweth S. F., McGraw E. A., 2009 Effective but costly, evolved mechanisms of defense against a virulent opportunistic pathogen in *Drosophila melanogaster. PLoS Pathog.* **5:** e1000385.

Zhang J., Marshall K. E., Westwood T., Clark M. S., Sinclair B. J., 2011 Divergent transcriptomic responses to repeated and single cold exposures in *Drosophila melanogaster. J. Exp. Biol.* **214:** 4021-9.
